# Global Performance Index for Integrated Management System: GPI-IMS

**DOI:** 10.3390/ijerph18137156

**Published:** 2021-07-04

**Authors:** Alessandro Silvestri, Domenico Falcone, Gianpaolo Di Bona, Antonio Forcina, Marco Gemmiti

**Affiliations:** 1 Department of Civil and Mechanics Engineering (DICEM), University of Cassino and Southern Lazio, 03043 Cassino, Italy; falcone@unicas.it (D.F.); dibona@unicas.it (G.D.B.); 2 Department of Engineering, Isola C4, Centro Direzionale Napoli, University of Naples “Parthenope”, 80143 Naples, Italy; antonio.forcina@uniparthenope.it; 3 INTIT Srl, 03013 Ferentino, Italy; marcogemmiti@gmail.com

**Keywords:** integrated budget, measure business performance, project management, risk assessment

## Abstract

Background: The present work starts from a literature review of the evolution of Integrated Management Systems (IMSs), considering different points of view and standards: quality, environmental, occupational health and safety, sustainability and social issues. Even if the benefits are possible, there is not a common approach and a clear link between the integration of management systems and business performance, in particular considering safety performance. Methods: The present study analyzes the application of Risk Assessment in order to realize the integration of management systems. The main objective is to provide a tool for an integrated evaluation of all company performances, starting from the definition of some Key Performance Indicators—KPIs—proposed for a particular case study, even if their choice is not the core of the paper. The assessment team members on the basis of their knowledge, experience and useful literature, could choose the right KPIs for the specific application, able to take a picture of the current state and to suggest a possible recommended action of improving. The proposed Risk Assessment approach is an integration of modern management techniques: Integrated Management System and Improving Cycle DMAIC. Results: The new method, called the Global Performance Index for Integrated Management System—GPI-IMS, has been applied to a real case study in the logistic field in order to evaluate its goodness and possible generalization. Conclusions: The proposed method allows to define the requirements that any company must have to perform the best. The role of the assessment team is very important to evaluate the global performance of the company and to suggest the corrective actions to be adopted.

## 1. Introduction

The integration of Management Systems has become a popular topic of research and practice; it has been discussed in the literature from different points of view and with different goals. Traditionally, the main investigated aspects are quality, environmental and occupational health and safety (OHS). The meaning itself of integration is often not so clear due to the absence of specific guidelines for the integration [1] (Wilkinson, G., 1999). Therefore, different approaches and models were proposed to realize an effective integration of different management systems, for example, ISO 9001 and ISO 14001 or OHSAS 18001 and SA 8000 [2,3,4] (Karapetrovic, S., 2002–2003). Additionally, Risk Analysis was used as a particular approach for the implementation of an Integrated Management System [5,6] (Labodová, A., 2004; Rocha, M., 2007). Many considerations are necessary to evaluate the benefits of integration, such as adequate resources, commitment and communication, etc. [7] (Zutshi, A., 2005). Another important issue is the culture incompatibility and employee hostility against new models and procedures [8] (Zeng, S.X., 2007). The levels of integration can be different, including the whole organization and all the involved stakeholders [9] (Jørgensen, T.H., 2006). Many experiences and studies were realized in different industrial sectors, both in western and eastern countries [10,11] (Zeng, S.X., 2008; Salomone, R., 2008), considering different ways, before ISO 9001 and then ISO 14001 or the opposite [12] (Karapetrovic, S., 2009); levels of integrations, all or some of the enterprise management systems [13] (Bernardo, M., 2009); and enterprise dimensions [14] (Santos, G., 2011). It is possible to observe an increasing interest in the concept of sustainable development; therefore, social issues were integrated with the traditional management of quality, environmental and occupational health and safety aspects [15,16,17,18] (Oskarsson, K., 2005; Asif, M., 2011; Ikram, M., 2019; Chaudhuri, A., 2019). Even if the literature review confirms the benefits and suggests recommendations for a correct integration of Management Systems [19] (Bernardo, M., 2015), there is not a common approach and a clear link between the integration of Management Systems and business performance [20,21] (Purwanto, A., 2020; Petrillo, A., 2018), in particular considering safety performance [22,23] (Falcone, D. 2007; Sarkheil, H., 2021).

The evolution of the economic context has highlighted the need to review traditional business models oriented not only to the interests of shareholders, redefining management processes from both an operational and strategic perspective. The present paper suggests a methodology to analyze the performance of any company that implements an Integrated Management System. In particular, in recent years, there has been a growing need to integrate the various reporting documents. The financial statements are no longer sufficient, as in the perspective of a “Triple Bottom Line” [24] (Elkington, J., 1998), considering economic prosperity, social equity and environmental protection as equally important. This need has been raised by public and private bodies, by the academic world as well as by financial operators,;in fact, it has brought in companies an increased awareness of the need to incorporate in their strategies a greater coherence and a wider vision. Therefore, in addition to the more strictly economic-financial aspects, it is essential to consider the prospective sustainability of the company, as well as the risks and opportunities linked to a wider range of internal and external factors. A new instrument was developed, called Integrated Balance Sheet, that allows analyzing a company’s performance through an Integrated Management System. The goal of the paper is to translate the moment of writing the above sheet into an opportunity to measure performance and to solve critical issues. Starting from the annual financial report, we moved to an integrated report based on sustainability, environmental and social issues and intangible assets, to define the performance analysis tool. Therefore, the Integrated Management System provides information not only on the amount of wealth produced, but also on how it was generated. It is possible to consider different points of view inside and outside the company. The overlapping of those different perspectives reveals common requirements and factors that are very relevant to measuring key activities for the success of any firm, impacting on different business aspects and consequently on the company performance.

## 2. The Integrated Management System—IMS

The Integrated Management System is a set of rules and procedures linked to a complete vision of company dynamics. In fact, it allows summarizing all the fundamental aspects for the success of the company in a single document. The main advantage of this system is that all business processes are analyzed simultaneously.

The proposed IMS focuses on five perspectives:

- Quality;
- Environment;
- Occupational Health and Safety;
- Ethics;
- Corporate Social Responsibility.

The management of activities in a business process according to the five above perspectives is able to guarantee a company success. Among them, Quality, Environment and Occupational Health and Safety are more frequently integrated, and their activities are considered important for product–process–people. Nowadays, due to the increase in attention to sustainable development goals and the shifting from the shareholder primacy model towards the stakeholder newest one, a new vision of the firm is arising based on the social contract theory that involves Ethics and Corporate Social Reasonability; a win–win approach both for companies and for their stakeholders.

Operational activities often impact different areas; therefore, a common vision is necessary. The proposed IMS, based on the definition and evaluation of a unique index, can allow improving the company performance reducing effort and suggest adequate corrective actions.

**Figure 1:**
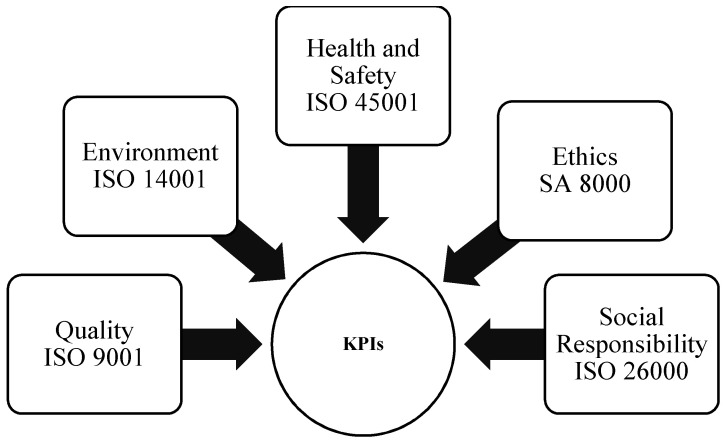
Integrated Management System scheme for defining Key Performance Indicators—KPIs.

Below is a brief overview of the international standards at the basis of an Integrated Management System (Figure 1):

- UNI EN ISO 9001: the international standard for Quality Management Systems (QMS), published by the International Organization of Standardization. The standard was last updated in 2015 and had to receive the approval of the majority of member states in order to be internationally recognized [25] (Standardization, UNI EN ISO 9001, 2015).
- UNI EN ISO 14001: the international standard for environmental issues, allowing:
  1. Managing the environmental impacts of its activities;
  2. Ensuring compliance with applicable legislation;
  3. Pursuing continuous improvement [26] (Standardization, UNI EN ISO 14001, 2015).
- UNI EN ISO 45001: the “Occupational Health and Safety Management Systems” standard is the first international standard to define minimum standards of good practices for the protection of workers worldwide. It establishes a framework to improve safety, reduce risks in the workplace and improve the health and well-being of workers, thus enabling increased health and safety performance [27] (Standardization, UNI EN ISO 45001, 2018) (It has replaced the old standard OHSAH 18001).
- SA 8000: the abbreviation SA stands for Social Accountability and identifies an international certification standard drawn up by the CEPAA Council of Economical Priorities Accreditation Agency and is aimed at certifying certain aspects of corporate management related to corporate social responsibility or Corporate Social Responsibility (CSR) [28] (Standardization, SA 8000, 2014). These factors are:
  1. tRespect for Human Rights;
  2. tRespect for Workers’ Rights;
  3. tProtection against the exploitation of minors;
  4. tAssurance of Safety and Health in the workplace. (Standardization, Social Accountability, 2014)
- UNI ISO 26000: an international standard that provides guidelines on Corporate Social Responsibility—CSR [29] (Standardization, UNI ISO 26000, 2010).

An IMS proposes a unified vision of the company management system through better visibility of common objectives, unique management and a single reference for documentation and data management, the optimization of resources, cost containment and better integration of skills. The tools used in these organizational schemes are common for the most part. An integrated vision of objectives is a prerequisite for implementing an Integrated Management System. In fact, it provides information not only on the amount of wealth produced but also on how it was generated. From the overlapping of many standards, common Key Performance Indicators—KPIs—emerge, that are very relevant for the success of any company. In the following, some KPIs are proposed for a particular case study in the logistical field, even if their choice is not the core of the paper, but only the followed procedure. The assessment team members, on the basis of their knowledge and experience, also referring to literature, could choose the right KPIs for the specific application able to take a picture of the current state and to suggest a possible recommended action of improvment.

The most common mistake is to consider management systems in a separate way, divided by responsibilities and objectives. In reality, they are mutually dependent, and it is essential to remember that an integrated approach is suggested by the standards themselves. In fact, UNI EN ISO 14001 quotes, “The integration of environmental disciplines into the organisation’s overall management system can contribute to the effective introduction of the environmental management system, as well as to its efficiency and clarity of roles” [26].

The Quality Management System is that part of an organisation’s management system that aims, with reference to quality objectives, to achieve results that adequately meet the needs, expectations and requirements of all stakeholders. Quality objectives are complementary to the organisation’s other objectives such as growth, financing, profitability, environment and health and safety in the workplace [30] (Standardization, UNI EN ISO 9000, 2015).

Thanks to this approach, we obtain information on the total performance of the processes, in order to eliminate problems and improve the organization and management of activities.

## 3. The Global Performance Index for Integrated Management System

The proposed performance analysis tool follows the UNI ISO 31000:2018 [31] (Standardization, UNI ISO 31000, 2018) risk assessment structure, and its name is the Global Performance Index for Integrated Management System—GPI-IMS.

The UNI ISO 31000:2018 [31] (Standardization, UNI ISO 31000, 2018) is a standard codified by the International Organization for Standardization that provides principles and generic guidelines on managing risks faced by organizations. Its recommendations can be customized to any organization and its context. The traditional Risk Index focuses on two aspects: Gravity (or Consequence) and Frequency (or Likelihood) to rank risks and consequently to define improvement actions (1).

Risk = Gravity *×* Frequency (1)

In introducing the new Index, the meaning of “risk” becomes “Performance loss”, and a global company view is considered instead of a single point of view. Consequently, Gravity and Frequency are, respectively, replaced by Relevance and Rating—Equation (2).

Performance = Relevance × Rating(2)

The first parameter considers the impact of General Requirements in terms of possible negative consequences on the company management, in particular on the five standards: quality, environment, health and safety at work, ethics and corporate social responsibility; instead, the second parameter considers the impact of specific aspects, valuable thanks to some Key Performance Indicators—KPIs. Therefore, the Global Index measures the possible impact in terms of performance loss, starting from the definition and evaluation of general and specific aspects. Ias the higher the Index is, the higher the possibility of negative consequences on the company performance if corrective actions are not programed as soon as the index value suggests.

The first value, called “Relevance”, does not depend on the particular company, but it is fixed in general, referring to the particular aspect (e.g., environmental or safety aspects). Instead, the second value, called “Rating”, is the result of a professional and objective evaluation by a Team of Experts. Both will be explained in a better way below.

**Figure 2:**
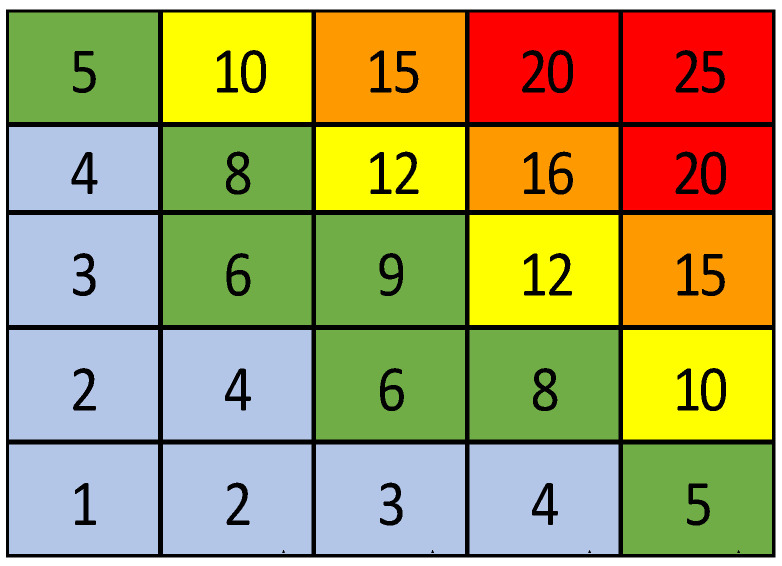
Global Performance Index matrix (the colors order blue-green-yellow-orange-red means an increasing GPI value and consequently a decreasing Performance value).

According to the fixed scales of judgment (range between 1 and 5 for both factors), the PPerformance Index range is from 1 to 25 (5 × 5 matrix). The proposed ranking for the GPI, starting from well-defined scales of judgment, considers values close to the maximum (Index of 25), as an insufficient performance; on the contrary, for values close to the minimum (Index of 1), we have excellent performance (Figure 2).

Therefore, the final value of GPI should be as low as possible, which means high performance and no intervention needed. This concept is simplified in Table 1 below, according to the UNI ISO 31000 approach, where the company performance decreases when the GPI value increases.

The structure of Global Performance Index is flexible and it can be divided into two main parts: a fixed part and a variable one. The first fixed part (Relevance) classifies all the requirements for an ideal organization of a particular sector of investigation. Instead, the second variable part (Rating) depends on the considered company.

**Figure 3:**
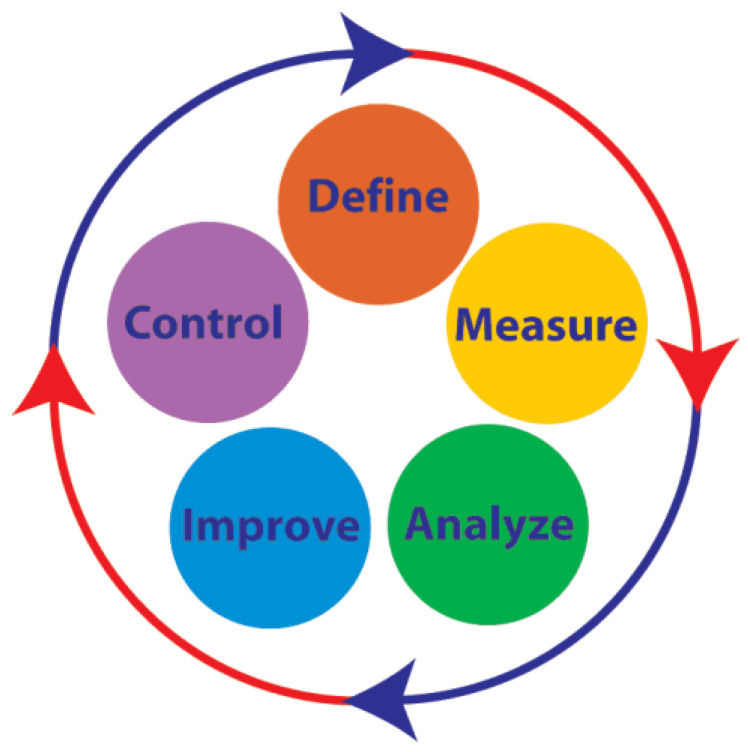
DMAIC cycle of improvement.

The procedure followed by the Team of Experts, called the Global Performance Index for Integrated Management System—GPI-IMS, is showed in the below case study; it translates the performance of a company into specific requirements and Key Performance Indicators—KPIs. The method steps are derived from the five phases of DMAIC: Define, Measure, Analyze, Improve and Control, the well-known cycle of improvement (Figure 3).

The analysis must not be conducted by a single person but by a Team of Experts who work together for the application of the proposed tool.

The composition of a Team of Experts representative of the company reality is at the basis of the success of the method because the GPI definition concerns the whole company and not only a part of it. The set of skills can easily consider all aspects in a complete and professional way, ensuring the correct application of GPI-IMS. In fact, the method cannot be standardized because it strongly depends on the conditions of the company and, above all, on how much the experts are able to analyze all the aspects.

**Figure 4:**
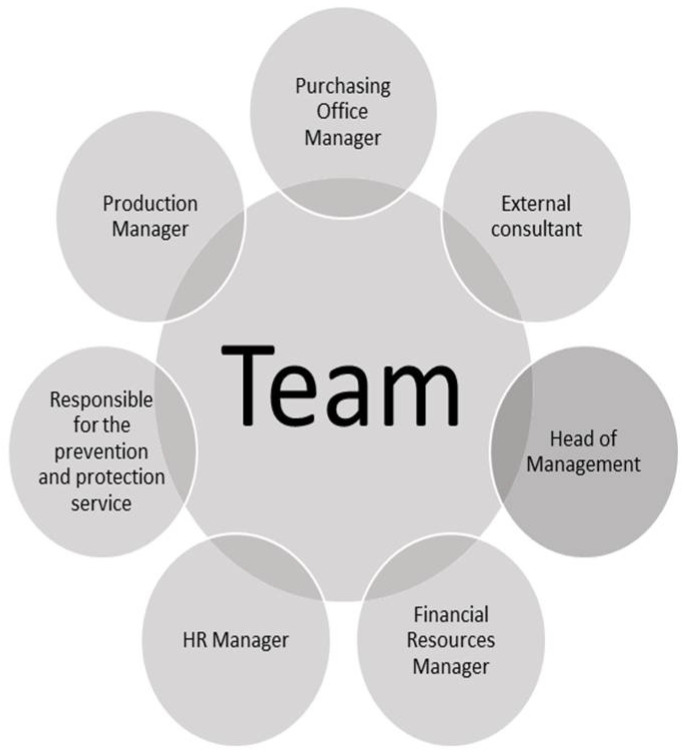
Team composition.

In the case study, the team was composed by an external consultant, a management manager, one of the financial resources, an HR manager, the Prevention and Protection Service manager, a production manager and the purchasing department manager. It is important to stress that the Team of Exerts should be heterogeneous to obtain a correct evaluation of the real performance. In Figure 4, the complete structure that each evaluation team should have is shown.

## 4. Case Study: Global Performance Index for Transport and Logistics

The case study considers the transport and logistic services, and the five steps are described.

### 4.1. Step 1—DEFINE

In the beginning, the analysis starts from the definition of the general requirements valid for any organization for the considered particular sector. Relevance of those defined requirements emerges from the overlapping of the five main international standards analyzed in the Integrated Management System section. In addition to the common points, other fundamental aspects could be analyzed to define the Performance framework. That first analysis resulted in 12 relevant requirements, and the level of relevance for each requirement was assessed. The weight 0 means no matching with the specific standard. Instead, high weights mean great importance for the considered standard of International Organization for Standardization (values from 1 to 5). Subsequently, the average of these weights was calculated to understand how Relevant each factor is—average value = Relevance factor, in Equation (2). Weights equal to 0 are not considered in the calculation of the mean value. In Table 2, the tool developed is shown for Relevance (Integrated Balance Sheet).

The last column is one of the two fundamental factors of GPI. Corresponding values are fixed for any type of company of the sector in which it operates. For this reason, the calculation of Relevance is valid in any subsequent Performance analysis; therefore, the starting point for the GPI-IMS application has been defined.

### 4.2. Step 2—ADAPT

This step is very important because it determines the success of the performance analysis. In fact, the GPI value depends on how the requirements identified in the first phase of the definition are adapted and how they are representative of the real conditions of the company. As previously mentioned, a heterogeneous team composition leads to a precise analysis of the company’s performance without making mistakes in the setup phase.

In the case study, some specific factors are pointed out. For each one of them, an Evaluation has been calculated by the Team of Experts, with a value between 1 and 5 associated with each Key Performance Indicator. A value of 1 corresponds to a very good evaluation, meaning that the company shows a very good condition for that particular KPI. A valueof 5 corresponds to an insufficient evaluation that needs to be improved. In Table 3, there is a summary of the scale of judgment used by the team for the Rating Evaluation.

At this point, for each factor, the team evaluates existing and measurable data. In fact, each qualitative characteristic is translated into a quantitative number. The translation of generic requirements into specific KPIs is the fundamental step of the proposed method and represents the variable part of the GPI-IMS method. Each company transforms the generic requirements into specific factors on the basis of its own data. Table 4 shows the Integrated Balance Sheet for the Ranking Evaluation for the considered transport and logistic company.

Each factor is studied by the team and a final assessment is realized, as mentioned above. For example, for the first factor called Non-compliance, the performance analysis is based on existing company data referred to as internal audits and claims. Then, the second factor, Employee engagement, is evaluated directly through an assessment by the Team of Experts, etc.

The last column indicates the Evaluation values used for the Performance calculation.

### 4.3. Step 3—MISURE

At this stage, the final global Performance Index of the company is calculated. The used formulation is the following, introduced before—Equation (2). 

Thanks to the Equation (2), we are able to calculate the Performance Index for each specific requirement identified in Step 2. The calculation is made by the team that multiplies the coefficients of Relevance, equal for all companies of the same field, and the Rating values specific for the considered company.

Therefore, in order to apply GPI-IMS, it is necessary that the Team of Experts translates the list of general requirements and specific factors into quantifiable and easy-to-understand numbers in the most professional and objective way. If the evaluation procedure does not comply with those guidelines, the analysis loses its meaning and is not representative of the real business conditions.

In the case study of the transport and logistic company, GPI-IMS is equal to 12.2. At this point, in the next final step, some corrective corrections are suggested.

### 4.4. Step 4—IMPLEMENT

**Figure 5:**
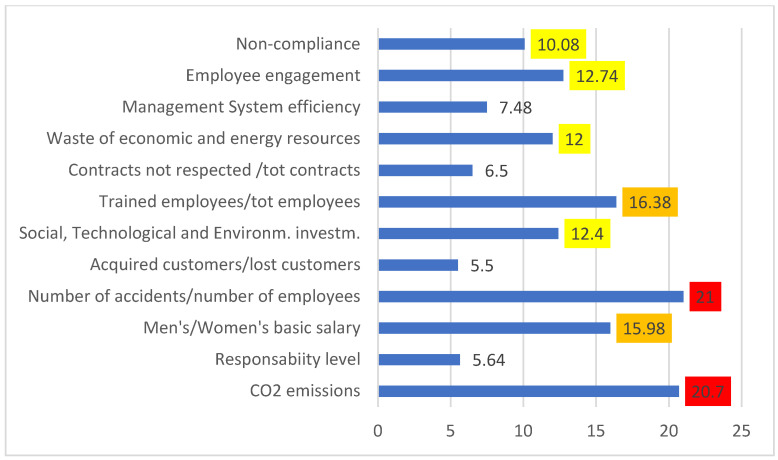
Process Performance (the colors order yellow-orange-red means an increasing GPI value and consequently a decreasing Performance value, Table 1).

In this step, the team establishes the corrective activities that have to be planned and implemented. The process follows the performance analysis backward: once the overall performance has been defined, the single performance of each specific factor is analyzed; thanks to this analysis, the critical points are highlighted. In Figure 5, the processes that generate very low Performance values are focused, signed in red. Then, we have the higher Performance processes in orange and finally those in yellow (i.e., lower GPI values mean higher Performance values). Green processes also need to be observed, but corrected in the long term; instead, blue ones are not critical (both not highlighted in the figure). Therefore, every critical factor for the company is solved through one or more corrective actions that the team thinks are useful for the analyzed company. Additionally, for this reason, the members of the team must be experts and must know the company under examination.

Corrective actions are divided into primary and secondary: for values close to the maximum (25), you have a critical performance. Consequently, processes that have very low values must be corrected earlier than others.

The primary corrective actions are as follows:

1. Health and Safety of Workers: to purchase new Personal Protective Equipment—PPE;
2. Reduction of CO_2_ emissions: to improve vehicle maintenance; to purchase 15 Hybrid vehicles;
3. Employee Training: new planning and increased annual training;
4. Corporate Social Responsibility: better salary balance between men and women; optimization of turnover management.

The secondary corrective actions are as follows:

1. Employee Engagement: increased training hours; introduction of personal awards; making company data accessible at all levels;
2. Environmental, Technological and Social Investment: introduction of social initiatives; to update hardware and software devices; to purchase a 100 kW photovoltaic system;
3. Reduction of Resource Waste: adoption of the Integrated Management System and the proposed performance analysis tool;
4. Resolution of non-conformities: to increase the number of internal audits and training hours.

The team decided to use these corrective actions for the transport and logistic company. Those proposals must be approved by the company's higher management. Therefore, the technical control of the benefits that the team proposes for low Performance processes is carried out.

In the next section, this concept is clarified.

### 4.5. Step 5—CONTROL

This step is very important to understand if the performance analysis has led to a real and reliable result. The Total Benefits must be higher than the Total Costs necessary to obtain them. The mathematical formula to apply is simple:

(3) $$\frac{\mathrm{Total}\mathrm{Benefits}}{\mathrm{Total}\mathrm{Costs}}\geq 1$$

If the value of the ratio is much higher than 1, it means that the analysis and the solutions found are very good. On the contrary, if the result is less than 1, you have to repeat the analysis with greater accuracy. Additionally, in this step, the team plays an important role.

In the case study of the transport and logistic company, the following costs were identified:

- New materials and PPEs purchasing: to calculate how expensive it is to reduce the probability of injury, the team took data of the last 5 years about injuries and observed that the main cause of injury was unsuitable work clothing. Therefore, the team estimated an average cost of €70.00 for each of the 500 employees to buy new materials. The proposal solves the problem of injuries with a cost of €70.00 × 500 = €35,000.00;
- Hybrid vehicle purchasing: wheeled transport is the company’s core business and optimizing this process is very beneficial. This is why the team has proposed the purchasing of 15 new hybrid vehicles to reduce CO_2_ emissions and fuel consumption. Moreover, this investment is parallel to the purchasing of a photovoltaic system. The total cost is equal to €26,750.00 × 15 = €401,250.00;
- Photovoltaic plant purchasing: this investment is useful for several reasons. First of all, it allows new hybrid vehicles to be recharged without cost. Then, the produced energy is self-consumed and also sold with substantial revenue. The cost is equal to €420,000.00 for a nominal power of kW 100;
- Maintenance costs: on the basis of historical data, a cost of €18,250.00 has been calculated for maintenance activities.

These programed investments create the following benefits:

- Injury reduction: each year, an average of 96 accidents occurs, and the estimated cost for each of them is equal to €1302.08. The benefit estimated by the team, thanks to the accidents reduction, is equal to 96 × €1302.08 = €125,000.00;
- Fuel saving: each vehicle travels km 51,033 per year. The company has 498 vehicles, but the saving is calculated only for the purchased 15 vehicles. Considering the average price of fuel and its saving, the total benefit is of €22,200.00;
- Energy saving: thanks to the purchasing of the photovoltaic system, an energy saving of €115,000.00 is possible and selling it a revenue of €447,657.00 is expected with a total benefit of €562,657.00;
- New potential contracts: finally, the company growth estimated with the previous interventions is equal to €870,000.00.

Therefore, the ratio (3) gives a value greater than 1, in particular, equal to 1.8 Equation (4), that means a good result in using the performance analysis tool.

(4) $$\frac{125,000+22,200+115,000+447,657+870,000}{35,000+401,250+18,250+420,000}=1.8\geq 1$$

In addition, the corrective actions have affected the real critical processes and improved the company’s overall performance.

## 5. Conclusions

In the present work, an integrated methodology, called the Global Performance Index for Integrated Management System, has been proposed and then applied to a transport and logistic company. The method allows monitoring the fundamental KPIs, linked to business activities relevant for any company success. First of all, a general approach was proposed with an integrated logic to summarize the company’s performance, applicable to any organization, and then tested in a specific real case. Thanks to GPI-IMS, it is possible to suggest and to adopt in time the right corrective actions to maximize the company performance, acting on the key business activities linked to the five standards: Quality, Environment, Health and Safety at Work, Ethics and Corporate Social Responsibility

Through the experimentation of the method of performance analysis, it has been understood that companies can no longer look exclusively at the economic result in operational terms because they are surrounded by other factors of influence that interfere with the same economic result. To ignore those factors negatively affects the current and future performance of the company itself. The intent of the proposed method is to offer companies an integrated approach that can analyze all the structural and operational aspects in order to return a numerical value through which to evaluate the company performance. Thanks to the proposed method, it is easy to identify which are the company aspects that need improvements or corrections, in order to manage the system consciously and in compliance with legal obligations.

The initial structure of the proposed methodology is general, but its application could be customized to the company under analysis, as each company differs from the others. In fact, in the case study, some of the proposed corrective actions have generated measurable effects in the short term, while others will be quantified in the medium–long term because they are more difficult to adopt. The critical aspect is that the method of performance analysis must be applied by a Team of Experts, as heterogeneous as possible.

In the future, the tool will be integrated with other management instruments in order to support companies in improving their performance.

## Figures and Tables

**Table 1:** Global Performance Index—GPI values and Performance range.

GPI	Performance	Corrective Action
GPI < 5	Very High	Corrective actions not to be planned immediately
5 ≤ GPI < 10	High	Corrective actions to be planned in the long-medium term
10 ≤ GPI < 15	Average	Corrective actions to be planned in the medium–short term
15 ≤ GPI < 20	Low	Corrective actions to be planned urgently
20 ≤ GPI ≤ 25	Very Low	Corrective actions needed immediately

GPI—Global Performance Index.

**Table 2:** Integrated Balance Sheet for Relevance.

General Requirements	ISO 9001 [25]	ISO 14001 [26]	ISO 26000 [29]	SA 8000 [28]	ISO 45001 [27]	Relevance
Compliance with Legislative Decree 81/2008 [32]	2	4	4	4	4	3.6
Correct functioning of the Management System	5	2	2	2	2	2.6
Ease of work management	5	2	1	1	2	2.2
Purchase at the best quality:price ratio	3	5	1	1	2	2.4
Compliance with civil and contractual regulations	4	3	2	2	2	2.6
Training	3	5	4	4	5	4.2
Environmental, social and technological impact	4	4	4	4	4	4
Customer satisfaction and loyalty	5	0	0	0	0	5
Health and Safety at work	3	4	4	5	5	4.2
Corporate Social Responsibility	0	0	5	5	4	4.7
Ethics	0	0	5	5	4	4.7
Environmental impact regulation	4	5	0	0	0	4.5

**Table 3:** Rating scale of judgment.

Rating Value	Judgment
1	Excellent
2	Good
3	Sufficient
4	Mediocre
5	Insufficient

**Table 4:** Integrated Balance Sheet for Ranking.

Specific Factors	Key Performance Indicator				Evaluation
Non-compliance	Environment internal audit 2	Quality internal audit 21	Customer claims 4	Total 27	2.8
Employee engagement	Team evaluation				4.9
Management System efficiency	Number of compliant orders 2999		Tot managed orders 12,645	Split ratio 0.237	3.4
Waste of economic and energy resources	Electricity 1.959 kW/man	Tyres 3.52 n/vehicle	Paths 51,033 km/vehicle	Fuel 4470 L/vehicle	5
Contracts not respected/tot contracts	Not respected Trained		Tot 15	Split ratio 0.2	2.5
Trained employees/tot employees	395		Tot 698	Split ratio 0.566	3.9
Social, Technological and Environment investment	Social €10,240	Technological €12,450	Environmental €500	Total €31,190	3.1
Acquired customers/lost customers	Acquired 19		Lost 12	Split ratio 1.583	1.1
Number of accidents/number of employees	Accidents 216		Employees 698	Split ratio 0.309	5
Men’s/Women’s basic salary	Men €1653		Women €1276	Split ratio 1.295	3.4
Responsibility level	Team Evaluation				1.2
CO_2_ emissions	CO_2_ emissions 6,254,738 g/vehicle		Paths 51,033 km/vehicle	Split ratio 122 g/km	4.6

## Data Availability

The data and code presented in this study are available on reques from the author.
